# Relationship Between Perceived Stress Level, Psychological Flexibility, Depression, and Anxiety in Patients with Acute Myocardial Infarction

**DOI:** 10.3390/medicina61071139

**Published:** 2025-06-25

**Authors:** Esra Polat, Şükrü Çiriş, Zekiye Çelikbas, Afnan Chaudhry

**Affiliations:** 1 Department of Cardiology, Gaziantep City Hospital, Gaziantep 27470, Turkey; dr.sukru06@hotmail.com; 2 Department of Psychiatry, Gölhisar State Hospital, Burdur 15400, Turkey; zekiyecelikbas@gmail.com; 3 Department of Internal Medicine, Phoenixville Hospital, Phoenixville, PA 19460, USA; afnan.chaudhry@towerhealth.org

**Keywords:** acute myocardial infarction, depression, psychological flexibility, anxiety

## Abstract

*Background and Objectives*: Stress and type A personality are known to be risk factors for the development of acute myocardial infarction (AMI), and depression is both a risk factor for AMI and a prognostic factor. In this study, we aimed to evaluate the relationship between psychological flexibility, perceived stress level, depression, and anxiety in AMI patients. *Material and Methods*: The study included 89 patients with a diagnosis of AMI and 89 volunteer participants with no previous history of coronary angiography and no diagnosis of AMI. Patients were evaluated with the Acceptance and Action Questionnaire (AAQ)-II, the Hospital Anxiety and Depression Scale (HADS), and the Perceived Stress Scale (PSS)-14. *Results*: A strong positive statistically significant correlation was found between the Perceived Stress Scale score and HAD-II (r = 0.697 *p* < 0.001), HAD-Anxiety (r = 0.715 *p* < 0.001), and HAD-Depression (r = 0.657 *p* < 0.001) scores. A statistically significant moderate positive correlation was found between the HAD-Depression Scale and HAD-Anxiety (r = 0.593 *p* < 0.001) and AAQ-II (r = 0.530 *p* < 0.001) scores. A strong positive statistically significant correlation was found between the HAD-Anxiety Scale and AAQ-II (r = 0.809 *p* < 0.001) scores. *Conclusions*: Investigation of psychological flexibility, anxiety, and depression in AMI patients with scales such as AAQ-II, PSS-14, and HADS may help early diagnosis and treatment of individuals at risk for psychiatric disorders.

## 1. Introduction

Acute myocardial infarction (AMI) is a heart disease characterized by myocardial necrosis caused by acute and prolonged ischemia of a coronary artery, which is commonly seen in the community and may result in death [1]. Smoking, hyperlipidemia, diabetes mellitus, hypertension, stress, and type A behavior, defined as ambition, determination, impatience, and a high desire for success, have been identified as risk factors for AMI, and it has been observed that stress and type A behavior are more common in young AMI patients [2,3,4,5,6].

The relationship between heart disease and psychiatric disorders has long been known. The risk of sudden cardiac death, which often occurs after the diagnosis of heart disease or life-threatening conditions related to heart disease, often causes anxiety and depression. The incidence of depressive symptoms in patients with AMI is 30–40%, while the incidence of major depression is 15–20% [7,8]. Many studies have shown that depression is both an important risk factor for AMI and a predictor of poor prognosis after AMI [9].

Psychological flexibility is defined as the ability to act in accordance with the individual’s targets and values in the face of distressing events, without unnecessary defense, as the situation allows, or to change them and not avoid the thoughts and feelings contained in that moment. This stance includes processes such as making appropriate choices and taking action even in the presence of challenging and painful memories, thoughts, emotions, or feelings [10]. Variables affected by psychological flexibility, such as depression, anxiety, and stress, have been shown to adversely affect cardiovascular risk and prognosis through biological mechanisms such as hypertension and blood pressure reactivity to stress and behavioral pathways such as decreased healthy behaviors [11].

To the best of our knowledge, there is no research that evaluates the relationship between psychological resilience, perceived stress level, depression, and anxiety in AMI patients. The hypotheses we established while performing this study were (i) In AMI patients, perceived stress, depression, and anxiety levels will be higher than in the control group, and psychological flexibility level will be lower; (ii) Perceived stress, depression, and anxiety levels will be negatively correlated with psychological flexibility; (iii) Psychological flexibility and perceived stress level will have a predictive role in depression and anxiety.

## 2. Material and Methods

### 2.1. Study Design and Settings

Institutional ethics committee approval was obtained from the Non-Interventional Ethical Clinical Research Ethics Committee.

This study was conducted in a hospital with a coronary angiography center and 24/7 primary percutaneous intervention.

### 2.2. Selection of the Participants

Patients aged 18 years and over who were diagnosed with AMI between 15 June 2022 and 15 August 2022 and who agreed to be included in this study and volunteers without AMI and without a history of coronary angiography were included in this study.

### 2.3. Measurements and Outcomes

Patients diagnosed with AMI were evaluated with the following assessment tools before discharge from the hospital and volunteers in the control group.

#### 2.3.1. Sociodemographic and Clinical Data Collection Form

A form was used to evaluate the sociodemographic and clinical characteristics of the patients, including age, gender, educational status, economic status, history of previous AMI, comorbid medical status, and body mass index. Patients’ regular medicine use due to any chronic illness and also their use of psychological drugs were examined.

#### 2.3.2. Acceptance and Action Questionnaire (AAQ)-II

This form was developed to measure the differences between individuals in psychological rigidity, which is one of the main dimensions of Acceptance and Commitment Therapy. It consists of seven questions and is of a single size. It is scored on a seven-point Likert scale. As the score obtained from the questionnaire increases, psychological rigidity and experiential avoidance increase, and psychological flexibility decreases [12]. The adaptation study of the scale in our country was carried out by Yavuz et al. Exploratory and confirmatory factor analyses were performed. Cronbach’s alpha consistency coefficient was found to be 0.84, and test–retest reliability was found to be r = 0.85 [13].

#### 2.3.3. Perceived Stress Scale (PSS)-14

It was developed by Cohen, Kamarck and Mermelstein (1983) [14]. Consisting of a total of 14 questions, the PSS is designed to measure the extent to which certain situations in a person’s life are perceived to be stressful. Participants rate each question on a 5-point Likert-type scale ranging from ‘Never (0)’ to ‘Very often (4)’. The 7 items with positive expressions are reverse scored. The scores of PSS-14 range from 0 to 56, and a high score indicates that the stress perception of the person is excessive. Turkish validity and reliability study was performed, and the internal consistency coefficient was found to be 0.84, and the test–retest reliability coefficient was found to be 0.87 [15].

#### 2.3.4. The Hospital Anxiety and Depression Scale (HADS)

The HADS is a Likert-type self-assessment scale. It consists of a total of 14 questions, seven of which investigate symptoms of depression and seven of which investigate symptoms of anxiety. The scale is a screening test to identify risk groups by screening anxiety and depression in patients with physical illness in a short time. The HADS scale was developed by Zigmond and Snaith [16]. The validity and reliability studies of the Turkish form were performed by Aydemir et al. Cronbach’s alpha coefficient was found to be 0.8525 for the anxiety subscale and 0.7784 for the depression subscale. The cut-off points were found to be 10 for the anxiety subscale and 7 for the depression subscale [17].

### 2.4. Statistical Analysis

Statistical Package for the Social Sciences (SPSS) version 25.0 program was used for data analysis. Descriptive data on sociodemographic and clinical information of the participants were given as *n* (%), mean ± SD tables.

These data were analyzed for normality assumptions, and Kolmogorov–Smirnov values were determined as *p* > 0.05. Pearson correlation analysis, one of the parametric tests, was performed to determine the relationship between scale scores according to the MI groups. In order to determine whether there is a significant difference between the scales according to the AMI groups and their education status, independent samples t-test and ANOVA test were applied. In case of a significant difference between the groups, the LSD test, one of the post-hoc tests, was used to determine which groups the significance was between. The Chi Square test or Fisher’s Exact test was used for the comparison of categorical variables. Finally, linear regression analyses were given for the prediction of the Fear of Death scale by the AAQ-II, HAD-Anxiety, and HAD-Depression scales. *p* < 0.05 was considered statistically significant.

## 3. Results

This study included 178 participants, including 89 patients with MI and 89 control volunteers. The mean age of the participants was 46.02 ± 15.54 years. The mean BMI of the patients with MI was statistically significantly higher than that of the control group (29.7 ± 5.04 vs. 26.25 ± 4.16, *p* < 0.001) (Table 1).

A statistically significant difference was observed between the MI group and the control group in terms of marital status (*p* < 0.001), educational status (*p* < 0.001), and employment status (*p* < 0.001) (Table 1).

When we examined Table 2, a statistically significant difference was observed between the MI group and the control group in terms of the presence of a history of medical illness (*p* < 0.001), presence of DM (*p* < 0.001), presence of HT (*p* < 0.001), presence of CAD (*p* < 0.001), and regular medication use (*p* < 0.001).

When the psychiatric disease and addiction status of the participants were analyzed, no statistically significant difference was observed between the MI group and the control group in terms of previous psychiatric diagnosis (*p* = 0.464), whereas a statistically significant difference was observed between the MI group and the control group in terms of smoking (*p* < 0.001) (Table 3).

In Table 4, when the total and sub-dimension scores of the scale were compared between the MI group and the control group, a statistically significant difference was observed between the two groups with the HAD depression scores (*p* = 0.002). The HAD depression scores of the group with MI were higher.

According to the correlations between the AAQ-II, HAD-Anxiety, HAD-Depression, and Perceived Stress Scale scores of patients who had MI, strong positive statistically significant relationships were found between the Perceived Stress Scale score and AAQ-II (r = 0.697 *p* < 0.001), HAD-Anxiety (r = 0.715 *p* < 0.001), and HAD-Depression (r = 0.657 *p* < 0.001) scores. Statistically significant moderate positive correlations were found between the HAD-Depression Scale and HAD-Anxiety (r = 0.593 *p* < 0.001) and AAQ-II (r = 0.530 *p* < 0.001) scores. A strong positive statistically significant correlation was found between the HAD-Anxiety Scale and AAQ-II (r = 0.809 *p* < 0.001) scores (Table 5).

The correlation between the relationship between the AAQ-II, HAD-Anxiety, HAD-Depression, and Perceived Stress Scale scores of the control group participants showed a statistically significant positive relationship between the Perceived Stress Scale score and the AAQ-II (r = 0.575 *p* < 0.001), HAD-Anxiety (r = 0.634 *p* < 0.001) and HAD-Depression (r = 0.466 *p* < 0.001) scores. A low statistically significant positive correlation was found between the HAD-Depression Scale and HAD-Anxiety (r = 0.480 *p* < 0.001) and AAQ-II (r = 0.446 *p* < 0.001) scores. A strong positive statistically significant correlation was found between the HAD-Anxiety Scale and AAQ-II (r = 0.725 *p* < 0.001) scores (Table 6).

A statistically significant difference was found between HAD-Depression scores and educational status (*p* = 0.005) when AAQ-II, HAD-Anxiety, HAD-Depression, and Perceived Stress scores were compared according to educational status in patients who had MI. HAD-Depression scores were observed to be higher in graduates below high school (Table 7).

In the control group participants, when the scores of the AAQ-II, HAD-Anxiety, HAD-Depression, and Perceived Stress were compared according to educational status, no statistically significant difference was found between the scores of the AAQ-II (*p* = 0.174), HAD-Anxiety (*p* = 0.306), HAD-Depression (*p* = 0.834), and Perceived Stress Scale (*p* = 0.804) and educational status (Table 8).

Table 9 shows the results of the linear regression analyses related to the prediction of the Perceived Stress Scale by the AAQ-II, HAD-Anxiety, and HAD-Depression Scales in patients with MI. According to the model results, it was concluded that the AAQ-II, HAD-Anxiety, and HAD-Depression Scales significantly predicted the Perceived Stress Scale (R = 0.791, R^2^ = 0.612, F = 47.124, *p* < 0.001). According to these results, Perceived Stress Scale scores increased significantly as the AAQ-II scores increased (Beta = 0.349, t = 2.589, *p* = 0.012), Perceived Stress Scale scores increased significantly as HAD-Anxiety scores increased (Beta = 0. 721, t = 2.327, *p* = 0.022), and Perceived Stress Scale scores increase significantly as HAD-Depression scores increased (Beta = 0.908, t = 4.058, *p* < 0.001).

## 4. Discussion

The aim of our study was to explore the relationship between psychological flexibility, perceived stress, anxiety, and depression and their potential predictive roles in acute myocardial infarction (AMI) patients. The main findings of this study were depression was higher in patients following acute myocardial infarction, but not anxiety [18,19]. The level of perceived stress correlates with the severity of depression and anxiety [20]. Psychological flexibility inversely correlates with perceived stress, anxiety, and depression.

Previous research reveals a complex relationship between depression and cardiovascular disease, with depression acting as both a risk factor and a consequence. A meta-analysis of 26 studies involving 1,957,621 individuals showed depression increased the risk of myocardial infarction by 28% [18]. Similarly, depression following an AMI can lead to poorer clinical outcomes. Henderson et al. found patients diagnosed with depression following myocardial infarction were three times more likely to be hospitalized for cardiovascular-related issues within two years [19]. Depression may influence cardiovascular disease through multiple mechanisms. It may promote unhealthy behaviors that promote traditional risk factors and reduce adherence to treatment. Additionally, depression may directly impact physiological systems, leading to autonomic dysfunction, metabolic syndrome, inflammation, and platelet dysfunction. Additionally, medications commonly used after AMI, such as beta-blockers and statins, may be linked to an increased risk of depression [20]. Although screening for depression is not yet a standard of care following AMI, strategies to improve early detection and management may improve cardiovascular outcomes.

Elevated levels of perceived stress are also associated with the development of coronary artery disease and, subsequently, worse clinical outcomes, likely through mechanisms similar to those involved in depression [21]. Narvaez et al. found that higher baseline levels of perceived stress are linked to increased depression, perhaps representing a modifiable risk factor [22]. Our study demonstrates a strong association between perceived stress and the severity of depression and anxiety in both control and AMI patients. Therefore, evaluating stress levels and initiating early interventions may help prevent mood disorders and enhance clinical outcomes after AMI. In a retrospective study of 61 patients following percutaneous coronary intervention, Gu et al. showed that mindfulness-based stress reduction significantly decreased PSS and HADS scores, improved blood pressure, and reduced major adverse cardiovascular events within three months of the intervention [23].

Psychological flexibility describes the ability to navigate through challenges and is theorized to be the mediator between perceived stress and depression or anxiety [24]. In our study, reduced psychological flexibility was associated with higher PSS and HADS scores, with stronger correlations to anxiety and depression in AMI patients. This indicates individuals recovering from AMI may experience greater emotional challenges, leading to increased stress and depressive symptoms, and highlights the potential value of interventions aimed at improving psychological flexibility. Acceptance and Commitment Therapy (ACT) aims to increase psychological flexibility in accordance with personal values by accepting negative thoughts rather than fighting them and developing an awareness of them through mindfulness-based techniques. A systematic review of 16 studies by Zhang et al. found that ACT effectively improved quality of life and self-care while reducing anxiety and depression in individuals with cardiovascular disease [25].

An additional important observation in our study was the relationship between educational status and depression in AMI patients. Individuals with lower education had higher depression scores following AMI, consistent with existing evidence that associates lower education levels with poorer mental health outcomes [26]. This finding emphasizes the need for an individualized mental health approach that takes into account socio-economic factors, such as educational background, to ensure that all patients receive the appropriate psychological support during their recovery.

Our study does have several limitations. The most important limitation was that the two groups were not propensity-matched for comorbidities, such as diabetes, hypertension, obesity, or pre-existing coronary artery disease, perhaps leading to confounding. The reason a matching control group could not be matched is that our hospital is the only state hospital in the province that performs primary percutaneous interventions. The majority of patients are those who have undergone coronary angiography, and those who volunteered for this study in the group without a history of coronary angiography were younger patients. The cross-sectional design of the study further limits the ability to infer causality. A propensity-matched longitudinal study would be beneficial to more directly determine the temporal relationship between perceived stress, psychological flexibility, anxiety, and depression in AMI patients. Additionally, our findings were confined to a single center and may not be generalizable to other hospital and cultural settings. Moreover, the patient cohort who agreed to participate in this study may possess certain predispositions, which may introduce a selection bias.

## 5. Conclusions

Following acute myocardial infarction, patients often face substantial emotional challenges, with depression emerging as a common consequence that negatively impacts both quality of life and clinical outcomes. Our study highlights the intricate relationship between perceived stress, psychological flexibility, anxiety, and depression. While psychological flexibility may influence how individuals cope with stress, our findings suggest that, ultimately, perceived stress is strongly associated with the severity of depression and anxiety symptoms. Incorporating routine mental health screening after AMI using validated instruments such as the AAQ-II, PSS-14, and HADS may enable early identification of at-risk individuals and facilitate interventions, such as ACT, before clinical depression develops. However, further research is still required to better understand the temporal relationship between perceived stress, psychological flexibility, anxiety, and depression post-AMI and to evaluate the long-term impact of psychological interventions on both mental health and cardiovascular outcomes.

## Figures and Tables

**Table 1:** The comparison of demographic variables according to MI groups.

	All Participants (*n* = 178)	MI Group		*p*
		Patient with MI (*n* = 89)	Control (*n* = 89)	
**Age (Year)**, Mean ± SD	46.02 ± 15.54	57.04 ± 12.31	35.0 ± 9.41	**<0.001 ^a^**
**Gender**, *n* (%)				
Male	116 (65.2)	65 (73)	51 (57.3)	**0.028 ^b^**
Female	62 (34.8)	24 (27)	38 (42.7)	
**BMI (kg/m^2^)**, Mean ± SD	27.81 ± 4.86	29.37 ± 5.04	26.25 ± 4.16	**<0.001 ^a^**
**BMI Group**, *n* (%)				
Normal	52 (29.2)	14 (15.7)	38 (42.7)	**<0.001 ^b^**
Overweight	82 (46.1)	43 (48.3)	39 (43.8)	
Obese	44 (24.7)	32 (36)	12 (13.5)	
**Marital status**, *n* (%)				
Married/living together	128 (71.9)	72 (80.9)	56 (62.9)	**<0.001 ^b^**
Single	29 (16.3)	0 (0.0)	29 (32.6)	
Other (Widowed/separated)	21 (11.8)	17 (19.1)	4 (4.5)	
**Education Status**, *n* (%)				
Under high school	50 (28.1)	50 (56.2)	0 (0)	**<0.001 ^b^**
High school	42 (23.6)	30 (33.7)	12 (13.5)	
Undergraduate and higher	86 (48.3)	9 (10.1)	77 (86.5)	
**Working Status**, *n* (%)				
Working	134 (75.3)	45 (50.6)	89 (100)	**<0.001 ^b^**
Not working	44 (24.7)	44 (49.4)	0 (0)	

^a^ Independent samples *t*-test, ^b^ Pearson Chi Square test, *p* < 0.005.

**Table 2:** Comparison of various disease variables according to MI groups.

	All Participants (*n* = 178)	MI Group		*p*
		Patient with MI (*n* = 89)	Control (*n* = 89)	
**Medical disease**, *n* (%)				
No	97 (54.5)	24 (27)	73 (82)	**<0.001 ^b^**
Yes	81 (45.5)	65 (73)	16 (18)	
**DM**, *n* (%)				
No	144 (80.9)	58 (65.2)	86 (96.6)	**<0.001 ^b^**
Yes	34 (19.1)	31 (34.8)	3 (3.4)	
**HT**, *n* (%)				
No	123 (69.1)	40 (44.9)	83 (93.3)	**<0.001 ^b^**
Yes	55 (30.9)	49 (55.1)	6 (6.7)	
**CAD**, *n* (%)				
No	147 (82.6)	58 (65.2)	89 (100)	**<0.001 ^b^**
Yes	31 (17.4)	31 (34.8)	0 (0)	
**HF**, *n* (%)				
No	175 (98.3)	86 (96.6)	89 (100)	0.246 ^c^
Yes	3 (1.7)	3 (3.4)	0 (0)	
**Renal Failure**, *n* (%)				
No	175 (98.3)	87 (97.8)	88 (98.9)	1.000 ^c^
Yes	3 (1.7)	2 (2.2)	1 (1.1)	
**Arrhythmia**, *n* (%)				
No	175 (98.3)	86 (96.6)	89 (100)	0.246 ^c^
Yes	3 (1.7)	3 (3.4)	0 (0)	
**Rheumatic disease**, *n* (%)				
No	174 (97.8)	88 (98.9)	86 (96.6)	0.621 ^c^
Yes	4 (2.2)	1 (1.1)	3 (3.4)	
**Regular drug use**, *n* (%)				
No	102 (57.3)	26 (29.2)	76 (85.4)	**<0.001 ^b^**
Yes	76 (42.7)	63 (70.8)	13 (14.6)	

^b^ Pearson Chi Square test, ^c^ Fisher’s Exact Chi Square test, *p* < 0.05. DM: Diabetes mellitus, HT: Hypertension, CAD: Coronary artery disease, HF: Heart failure.

**Table 3:** The comparison of psychiatric disease and addiction status of the participants according to MI groups.

	All Participants (*n* = 178)	MI Group		*p*
		Patient with MI (*n* = 89)	Control (*n* = 89)	
**History of psychiatric diagnosis**, *n* (%)				
No	140 (78.7)	68 (76.4)	72 (80.9)	0.464 ^b^
Yes	38 (21.3)	21 (23.6)	17 (19.1)	
**Depression**, *n* (%)				
No	156 (87.6)	78 (87.6)	78 (87.6)	1.000 ^b^
Yes	22 (12.4)	11 (12.4)	11 (12.4)	
**OCD**, *n* (%)				
No	177 (99.4)	89 (100)	88 (98.9)	1.000 ^c^
Yes	1 (0.6)	0 (0)	1 (1.1)	
**Anxiety**, *n* (%)				
No	163 (91.6)	79 (88.8)	84 (94.4)	0.177 ^b^
Yes	15 (8.4)	10 (11.2)	5 (5.6)	
**Behavioral disorders**, *n* (%)				
No	177 (99.4)	88 (98.9)	89 (100)	1.000 ^c^
Yes	1 (0.6)	1 (1.1)	0 (0)	
**Delirium**, *n* (%)				
No	177 (99.4)	88 (98.9)	89 (100)	1.000 ^c^
Yes	1 (0.6)	1 (1.1)	0 (0)	
**Addiction**, *n* (%)				
No	176 (98.9)	87 (97.8)	89 (100)	0.497 ^c^
Yes	2 (1.1)	2 (2.2)	0 (0)	
**Psychological drug use**, *n* (%)				
No	144 (80.9)	68 (76.4)	76 (85.4)	0.127 ^b^
Yes	34 (19.1)	21 (23.6)	13 (14.6)	
**Psychotherapy**, *n* (%)				
No	163 (91.6)	82 (92.1)	81 (91)	0.787 ^b^
Yes	15 (8.4)	7 (7.9)	8 (9)	
**Psychiatric hospitalization**, *n* (%)				
No	177 (99.4)	88 (98.9)	89 (100)	1.000 ^c^
Yes	1 (0.6)	1 (1.1)	0 (0)	
**Suicide attempt**, *n* (%)				
No	177 (99.4)	88 (98.9)	89 (100)	1.000 ^c^
Yes	1 (0.6)	1 (1.1)	0 (0)	
**Psychiatric illness in the family**, *n* (%)				
No	163 (91.6)	79 (88.8)	84 (94.4)	0.280 ^b^
Yes	15 (8.4)	10 (11.2)	5 (5.6)	
**Smoking/alcohol/drug use**, *n* (%)				
No	96 (53.9)	36 (40.4)	60 (67.4)	**<0.001 ^b^**
Yes	82 (46.1)	53 (59.6)	29 (32.6)	
**Smoking**, *n* (%)				
No	100 (56.2)	37 (41.6)	63 (70.8)	**<0.001 ^b^**
Yes	78 (43.8)	52 (58.4)	26 (29.2)	
**Alcohol**, *n* (%)				
No	169 (94.9)	87 (97.8)	82 (92.1)	0.168 ^c^
Yes	9 (5.1)	2 (2.2)	7 (7.9)	
**Drug use**, *n* (%)				
No	174 (97.8)	85 (95.5)	89 (100)	0.121 ^c^
Yes	4 (2.2)	4 (0.5)	0 (0)	

^b^ Pearson Chi Square test, ^c^ Fisher’s Exact Chi Square test, *p* < 0.05. OCD: Obsessive–compulsive disorder.

**Table 4:** Comparison of total and subscale scores in terms of MI groups.

	All Participants (*n* = 178)	MI Group		*p*
		Patient with MI (*n* = 89)	Control (*n* = 89)	
AAQ II Score, Mean ± SD	19.14 ± 8.47	19.02 ± 8.76	19.27 ± 8.24	0.846
HAD Anxiety, Mean ± SD	6.42 ± 3.92	6.37 ± 4.02	6.48 ± 3.86	0.849
HAD Depression, Mean ± SD	6.48 ± 3.58	7.3 ± 3.87	5.67 ± 3.10	**0.002**
Perceived Stress, Mean ± SD	21.35 ± 9.54	20.01 ± 10.42	22.7 ± 8.44	0.061

Independent samples *t*-test, *p* < 0.005.

**Table 5:** The relationships between the AAQ-II, HAD-Anxiety, HAD-Depression, and Perceived Stress Scale in patients with MI.

		AAQ-II	HAD-Anxiety	HAD-Depression	Perceived Stress
**AAQ-II**	r	1			
	*p*				
HAD-Anxiety	r	0.809 **	1		
	*p*	**<0.001**			
HAD-Depression	r	0.530 **	0.593 **	1	
	*p*	**<0.001**	**<0.001**		
Perceived Stress	r	0.697 **	0.715 **	0.657 **	1
	*p*	**<0.001**	**<0.001**	**<0.001**	

* Correlation is significant at 0.05 level (Pearson correlation test), ** Correlation is significant at 0.01 level (Pearson correlation test).

**Table 6:** The relationships between AAQ-II, HAD-Anxiety, HAD-Depression, and Perceived Stress Scale in patients without MI.

		AAQ-II	HAD-Anxiety	HAD-Depression	Perceived Stress
**AAQ-II**	r	1			
	*p*				
HAD-Anxiety	r	0.725 **	1		
	*p*	**<0.001**			
HAD-Depression	r	0.446 **	0.480 **	1	
	*p*	**<0.001**	**<0.001**		
Perceived Stress	r	0.575 **	0.634 **	0.466 **	1
	*p*	**<0.001**	**<0.001**	**<0.001**	

* Correlation is significant at 0.05 level (Pearson correlation test), ** Correlation is significant at 0.01 level (Pearson correlation test).

**Table 7:** Comparison of AAQ-II, HAD-Anxiety, HAD-Depression, and Perceived Stress Scale scores in patients with MI in terms of educational status.

	Education	*n*	Mean ± SD	F	*p*	* Post-Hoc
AAQ-II	(1) Under high school	50	20.44 ± 8.47	2.349	0.102	**-**
	(2) High school	30	18.17 ± 9.71			
	(3) Undergraduate and higher	9	14.00 ± 4.27			
HAD-Anxiety	(1) Under high school	50	7.06 ± 3.92	3.002	0.055	**-**
	(2) High school	30	6.03 ± 4.30			
	(3) Undergraduate and higher	9	3.67 ± 2.35			
HAD-Depression	(1) Under high school	50	8.32 ± 4.00	5.555	**0.005**	**1–2, 3**
	(2) High school	30	6.50 ± 3.37			
	(3) Undergraduate and higher	9	4.33 ± 2.55			
Perceived Stress	(1) Under high school	50	20.94 ± 11.54	0.989	0.376	-
	(2) High school	30	19.77 ± 9.23			
	(3) Undergraduate and higher	9	15.67 ± 6.54			

One Way ANOVA Test, Post-Hoc = LSD * *p* < 0.05 statistically significant.

**Table 8:** The comparison of AAQ-II, HAD-Anxiety, HAD-Depression, and Perceived Stress Scale scores in patients without MI in terms of educational status.

	Education	*n*	Mean ± SD	t	*p*
AAQ-II	High school	12	16.25 ± 7.41	−1.372	0.174
	Undergraduate and higher	77	19.74 ± 8.31		
HAD-Anxiety	High school	12	5.42 ± 4.25	−1.030	0.306
	Undergraduate and higher	77	6.65 ± 3.79		
HAD-Depression	High school	12	5.92 ± 4.38	0.215	0.834
	Undergraduate and higher	77	5.64 ± 2.89		
Perceived Stress	High school	12	21.92 ± 11.94	−0.253	0.804
	Undergraduate and higher	77	22.82 ± 7.86		

Independent samples *t*-test, *p* < 0.05 statistically significant.

**Table 9:** Linear regression analysis of AAQ-II, HAD-Anxiety, and HAD-Depression Scales predicting Perceived Stress Scale in patients with MI.

	B	SH	Beta	t	*p*	%95 CI	
						Lower	Lower
**(Constant)**	**2.151**	**1.811**		**1.188**	0.238	−1.450	5.753
AAQ-II	0.349	0.135	0.293	2.579	**0.012**	0.080	0.618
HAD-Anxiety	0.721	0.310	0.279	2.327	**0.022**	0.105	1.338
HAD-Depression	0.908	0.224	0.337	4.058	**<0.001**	0.463	1.352
**R = 0.791, R^2^ = 0.612, F = 47.124, *p* < 0.001**							

These significant variables explain approximately 61% of the total variance of the Perceived Stress Scale scores.

## Data Availability

Data used to support the findings of this study are available from the corresponding author upon reasonable request.
